# Evolution of *SHORT VEGETATIVE PHASE* (*SVP*) genes in Rosaceae: Implications of lineage‐specific gene duplication events and function diversifications with respect to their roles in processes other than bud dormancy

**DOI:** 10.1002/tpg2.20053

**Published:** 2020-09-17

**Authors:** Jinyi Liu, Min Ren, Hui Chen, Silin Wu, Huijun Yan, Abdul Jalal, Changquan Wang

**Affiliations:** ^1^ College of Horticulture Nanjing Agricultural University Nanjing Jiangsu 210095 China; ^2^ Yunnan Academy of Agricultural Sciences Flower Research Institute Kunming Yunnan 650200 China; ^3^ Shanghai Forestry Station Shanghai 200072 China

## Abstract

MADS‐box genes that are homologous to *Arabidopsis SHORT VEGETATIVE PHASE* (*SVP*) have been shown to play key roles in the regulation of bud dormancy in perennial species, particularly in the deciduous fruit trees of Rosaceae. However, their evolutionary profiles in Rosaceae have not yet been analyzed systematically. Here, The *SVP* genes were found to be significantly expanded in Rosaceae when compared with annual species from Brassicaceae. Phylogenetic analysis showed that Rosaceae *SVP* genes could be classified into five clades, namely, SVP1, SVP2‐R1, SVP2‐R2, SVP2‐R3 and SVP3. The SVP1 clade genes were retained in most of the species, whereas the SVP2‐R2 and SVP2‐R3 clades were found to be Maleae‐ and Amygdaleae‐specific (Both of the lineages belong to Amygdaloideae), respectively, and SVP2‐R1 was Rosoideae‐specific in Rosaceae. Furthermore, 10 lineage‐specific gene duplication (GD) events (GD1–10) were proposed for the expansion of *SVP* genes, suggesting that the expansion and divergence of Rosaceae *SVP* genes were mainly derived by lineage‐specific manner during evolution. Moreover, tandem and segmental duplications were the major reasons for the expansion of *SVP* genes, and interestingly, tandem duplications, a well‐known evolutionary feature of *SVP* genes, were found to be mainly Amygdaloideae‐specific. Sequence alignment, selection pressure, and *cis*‐acting element analysis suggested large functional innovations and diversification of *SVP* genes in different lineages of Rosaceae. Finally, the different growth cycle of *Rosa multiflora* and their novel expression patterns of *RmSVP* genes provided new insights into the functional diversification of *SVP* genes in terms of their roles in processes other than bud dormancy.

**Abbreviations**
DAM: dormancy associated MADS‐box
FT: FLOWERING LOCUS T
GD: gene duplication
GDR: Genome Database for Rosaceae
*Ka*
: nonsynonymous substitutions per nonsynonymous site
*Ks*
: synonymous substitutions per synonymous site
MADS: Mcm1‐Agamous‐Deficiens‐S
SOC1: SUPPRESSOR OF OVEREXPRESSION OF CONSTANS1
WGD: whole‐genome duplication.

## 1 INTRODUCTION

Winter is a major challenge for the reproductive success or even survival of most temperate plants. To adapt to the harsh environmental conditions of winter, plants have evolved appropriate strategies with highly dynamic mechanisms between species lineages (Bouche, Woods, & Amasino, 2017; Ream, Woods, & Amasino, 2012; Rohde & Bhalerao, 2007). Winter annuals such as Brassicaceae species and biennials have evolved a series of repression mechanisms to prevent reproductive development and avoid flower transition during winter without growth cessation. These repressive processes can be overcome by prolonged winter cold, very well‐known as vernalization (Bouche et al., 2017). However, perennials such as deciduous trees of Rosaceae, in particular, exhibit bud dormancy, a period between bud set in the autumn and budbreak in the spring with no visible growth, as a prominent strategy for survival and reproductive success under winter conditions (Falavigna, Guitton, Costes, & Andres, 2019; Rohde & Bhalerao, 2007). Like vernalization, bud dormancy release requires exposure to prolonged chilling temperatures; however, this chilling only restores the ability to grow but does not promote growth (Rohde & Bhalerao, 2007). This leads to a clue for the crucial differences between vernalization and bud dormancy release, which is further defined by the fact that vernalization occurs effectively in only actively dividing cells and dormancy is released by exposure to low temperatures after termination of cell division (Rohde & Bhalerao, 2007; Wellensiek, 1962, 1964). Currently, the regulatory pathways of vernalization in some model plants such as *Arabidopsis* have been well characterized. However, the mechanism of bud dormancy in perennial plants remains unclear and seems to be more diverse and complicated (Rohde & Bhalerao, 2007).

In Brassicaceae species, particularly in *Arabidopsis*, *FLOWERING LOCUS C* (*FLC*) is the key regulator of vernalization and can interact with *SHORT VEGETATIVE PHASE* (*SVP*) to negatively regulate *SOC1* and *FT* and suppress the transition of reproductive development and flowering (Aikawa, Kobayashi, Satake, Shimizu, & Kudoh, 2010; Amasino, 2010; Tao et al., 2012; Willmann & Poethig, 2011). Both genes are members of the MADS‐box transcription factor family and belong to the FLC and SVP subfamilies, respectively (Par̆enicová et al., 2003). However, no similar functions of FLC homologs have been reported in other plant lineages. Conversely, many studies have shown that *SVP* genes play crucial roles in the regulation of bud dormancy in perennial species (especially in the deciduous fruit trees of Rosaceae (Alexandre & Hennig, 2008; Falavigna et al., 2019; Rohde & Bhalerao, 2007; Wu et al., 2017), which is an economically important family worldwide (Longhi et al., 2014)). Particularly, the deletion of 6 SVP homologs in peach (*Prunus persica*), a well‐known *evergrowing* (*evg*) mutant, resulted in a complete lack of dormancy and were named as *DORMANCY ASSOCIATED MADS*‐*BOX* (*DAM*) genes (Bielenberg et al., 2008; Jimenez, Reighard, & Bielenberg, 2010; Kurokura, Mimida, Battey, & Hytonen, 2013; Rodriguez, Sherman, Scorza, Wisniewski, & Okie, 1994). Quantitative trait locus mapping and expression profiles of *DAM* genes in other *Prunus* species were shown to be highly dependent on the establishment, maintenance, and release of bud dormancy (Bielenberg et al., 2015; Fan et al., 2010; Leida, Conesa, Llacer, Badenes, & Rios, 2012). Furthermore, similar functions of *SVP* genes were also observed in other species of Rosaceae (Kurokura et al., 2013). For example, the expression profiles of apple *SVP* homologous genes, including *MdDAMa*, *MdDAMb*, *MdDAMc*, *MdSVPa*, and *MdSVPb*, were highly related to autumn growth cessation and bud dormancy maintenance. In addition, the overexpression of *MdSVPa* and *MdSVPb* resulted in budbreak delay and bud architecture changes (Wu et al., 2017). Similarly, the expression level of pear *PpMADS13‐1* was induced in dormancy establishment and then decreased concomitantly with dormancy release (Saito et al., 2013; Saito et al., 2015; Tuan, Bai, Saito, Ito, & Moriguchi, 2017). In Mei (*Prunus mume*), all *PmDAM* genes (*PmDAM1*–*PmDAM6*) were strongly repressed during prolonged cold exposure in winter and maintained at low levels till the release of endodormancy, and overexpression of *PmDAM6* in poplar (*Populus tremula* × *Populus tremuloides*) led to growth cessation and terminal bud set and bud endodormancy, even under favorable conditions (Sasaki et al., 2011). Interestingly, similar to the functional profiles of *FLC* genes and their homologs in Brassicaceae, some of the *DAM* genes, such as *DAM5* and *DAM6* in peach, could be suppressed by chilling temperatures and are inversely correlated with bud break rate (Jimenez et al., 2010), and those processes are also regulated by histone modifications and DNA methylation (de la Fuente, Conesa, Lloret, Badenes, & Rios, 2015; Leida et al., 2012; Rothkegel et al., 2017; Saito et al., 2015), as well as day length (Li, Reighard, Abbott, & Bielenberg, 2009) and plant hormones (Falavigna et al., 2019; Kurokura et al., 2013; Rinne et al., 2011; Tuan et al., 2017; Tylewicz et al., 2018), similar to *FLC* in Brassicaceae (Rios, Leida, Conejero, & Badenes, 2014). These results suggested that the biological functions of *SVP* genes, rather than *FLC* genes, might have been evolutionarily enhanced for bud dormancy in Rosaceae (Falavigna et al., 2019.

Bud dormancy is an adaptive process that allows perennial plants to survive the winter conditions of temperate climates (Falavigna et al., 2019; Paul, Rinne, & van der Schoot, 2014). The evolution of *SVP* genes, as the key regulators of this process, in Rosaceae is intriguing. Initially, the 6 *SVP*/*DAM* genes in peach (called the *evg* locus) were tandemly arranged at the end of chromosome 1 (Bielenberg et al., 2008). Similarly, the 6 homologs of *DAM* genes in Japanese apricot (*Prunus mume* Sieb. et Zucc.) were found to be tandemly arrayed on chromosome 2 (Sasaki et al., 2011). In addition, *DAM* genes in both apple and pear were found to have close chromosomal locations (Porto et al., 2016). The tandem compositions of *SVP* genes were also found in other perennial species, such as *Populus trichocarpa*, *Mimulus guttatus*, and *Phaseolus vulgaris* (Liu et al., 2018b). These results suggest that tandem duplication could be an evolutionary feature of *SVP* genes. Moreover, high collinearity or synteny relationships of *DAM* genes were identified between pear (*Pyrus communis* L.) and apple (*Malus domestica* Borkh.) genomes (Gabay et al., 2017), which indicates a recent whole‐genome duplication (WGD) event that specifically occurred in the pear and apple lineage (Xiang et al., 2017), suggesting that WGD may have contributed to the expansion of *SVP* genes in Rosaceae. Furthermore, evolutionary analysis of peach *DAM* genes showed a strong purifying selection mainly constrained by their functional divergence, and only a single codon, located in the C‐terminal region, was identified under significant positive selection (Jimenez, Lawton‐Rauh, Reighard, Abbott, & Bielenberg, 2009). These results yield the preliminary evolutionary features of *SVP* genes in Rosaceae. However, systematic evolutionary analysis of *SVP* genes in Rosaceae is still required.

Rosaceae is a moderately large angiosperm family in the order Rosales, with approximately 3000 species (Xiang et al., 2017) classified into 3 subfamilies: Rosoideae (∼2000 species), Amygdaloideae (∼1000 species), and Dryadoideae (∼30 species) (Potter et al., 2007). Most of the plants are economically important worldwide including not only many important fruits such as apple, pear, peach, strawberries, plum, cherry, and raspberry but also ornamental trees and flowers such as rose, meadowsweet, rowan, and hawthorn (Longhi et al., 2014; Xiang et al., 2017). The release of the genome sequences of 24 Rosaceae species covering 2 major lineages (https://www.rosaceae.org/) provided us the opportunity to study the comprehensive evolutionary profiles of *SVP* genes in Rosaceae. In this study, first, we performed a genome‐wide identification of *SVP* genes in the 24 Rosaceae species, covering the major subfamilies (Rosoideae and Amygdaloideae) of the order Rosales, and found that *SVP* genes were significantly expanded in Rosaceae. Second, we performed a systematic phylogenetic analysis to obtain the overall evolutionary picture of *SVP* genes in Rosaceae. Furthermore, we performed comparative analysis of the protein sequences and promoter regions of *SVP* genes to understand their functional diversification in different lineages of Rosaceae. Finally, we determined the expression of *SVP* genes in *Rosa multiflora* (seasonal flowering) and found novel regulatory mechanisms of *SVP* genes, other than bud dormancy. Our results provide comprehensive insights into the evolution and functional diversification of the *SVP* gene family in Rosaceae.

**Core Ideas**

- The *SVP* genes were significantly expanded in Rosaceae
- 10 lineage‐specific gene duplication (GD) events were proposed
- The tandem duplications were found to be mainly Amygdaloideae‐specific in Rosaceae
- Novel expression patterns of *SVP* genes were identified in *Rosa multiflora*

## 2 MATERIALS AND METHODS

### 2.1 Data sources and sequence retrieval

The genome data of most Rosaceae species were retrieved from GDR (https://www.rosaceae.org/). In addition, the genome data of 4 *Fragaria* species were downloaded from Strawberry GARDEN (http://strawberry-garden.kazusa.or.jp/), and the genome of *P. mume* was downloaded from NCBI/Genome (https://www.ncbi.nlm.nih.gov/genome). The *Rosa multiflora* genome was downloaded from a specific genome portal (http://rosa.kazusa.or.jp/index.html). Different versions of *Rosa chinensis* genome were downloaded from the specific genome portals (https://iris.angers.inra.fr/obh/ for V1 and https://lipm-browsers.toulouse.inra.fr/pub/RchiOBHm-V2 for V2) because we believed that the different versions are related to different homologous chromosomes. The latest version genome data of 9 Brassicaceae species and other species, including *Oryza sativa*, *Zea mays*, *Gossypium raimondii*, *Populus trichocarpa*, *Medicago truncatula*, *Glycine max* and *Cucumis sativus*, were downloaded from Phytozome v12 (https://phytozome.jgi.doe.gov/pz/portal.html) and *Vitis vinifera* (basal Rosids) were downloaded from CRIBI (http://genomes.cribi.unipd.it/).

### 2.2 Identification of *SVP* genes

First, all MADS‐box genes from the 24 Rosaceae, 9 Brassicaceae species, 2 Poaceae species, 2 other Malvidae species, 3 other Fabidae species and *Vitis vinifera* (basal Rosids), were identified using HMMER v3.0 (http://hmmer.org/), with 2 different HMM profiles for MADS‐box domains. One profile was obtained from a previous publication (Gramzow & Theissen, 2013), and the other SRF‐TF domain HMM profile was downloaded from the Pfam database (http://pfam.xfam.org/) (Liu et al., 2018). The K domain (PF01486), also downloaded from the Pfam database, was used to distinguish between the type I and type II MADS‐box proteins by using HMMER v3.0 software. Protein sequences of the type II MADS‐box genes were further verified using SMART (http://smart.embl-heidelberg.de/) and Pfam databases for integrity of the MADS‐box and K‐box domains. All verified type II MADS‐box proteins were subsequently used to construct a phylogenetic tree using FastTree software (http://www.microbesonline.org/fasttree/) with the JTT+CAT model based on full‐length sequences (Supplementary Figure S1) and IQTREE software (http://www.iqtree.org/) with the JTT+R8 model based on full‐length sequences (Supplementary Figure S2), and the subfamilies were defined on the basis of the phylogeny of the tree and classification of *Arabidopsis* counterparts. Finally, the SVP subfamily with a high bootstrap value (100) was selected for further analysis. For *SVP* gene identification from *Rosa multiflora*, three more *SVP* genes, *Rmu_sc0008851.1_g000013.1*, *Rmu_sc0005112.1_g000009.1*, *Rmu_sc0008447.1_g000001.1*, were identified without filtering with K‐box domain and were not included for evolution analysis.

### 2.3 Sequence alignment and phylogenetic analyses

The alignment software MAFFT v7.037b (Katoh & Standley, 2013) was used for multiple sequence alignments with the L‐INS‐I alignment strategy (most accurate), and maximum‐likelihood trees were constructed using both of the FastTree software with the JTT+CAT model (http://www.microbesonline.org/fasttree/) (Price, Dehal, & Arkin, 2009) and IQTREE with the JTT+R5 or JTT+R8 model (http://www.iqtree.org/). The phylogenetic trees were further visualized and edited using MEGA7 software (https://www.megasoftware.net/home) (Kumar, Stecher, & Tamura, 2016). In the phylogenetic trees, the bootstrap values that less than 50 were generally regarded as unreliable and were hid. The protein structures of each clade of the SVP proteins were generated using the Weblogo online program (http://weblogo.berkeley.edu/logo.cgi) (Crooks, Hon, Chandonia, & Brenner, 2004).

### 2.4 Synteny analysis and *Ka*/*Ks* determination

Firstly, gff3 files were downloaded from genome portals and formatted with local Python scripts, and all‐to‐all blast were executed using local blastall. Then, segmental/tandem duplication events were determined using MCScanX with default parameters (http://chibba.pgml.uga.edu/mcscan2/) in whole genome level for each species. Finally, segmental/tandem duplication events for *SVP* genes were extracted for our analysis using R program. Nonsynonymous (*Ka*) and synonymous (*Ks*) nucleotide substitution values and their ratios (*Ka*/*Ks*) between different clades of *SVP* genes were calculated by using pamlX v1.3.1 (Xu & Yang, 2013).

### 2.5 Analysis of positive selection

EasyCodeML software (https://github.com/BioEasy/EasyCodeML) (Gao et al., 2019) was used to evaluate the evolutionary selection in the *SVP* family in Rosaceae. First, the short sequences and sequences with large un‐aligned regions were removed manually (Supplementary Figure S3). Then, both branch model (BM) and branch site model (BSM) were used to detect the selection constraints for each clade or subclades in phylogenetic trees, and the likelihood ratio test was performed with default parameters to evaluate the significance. Bayes Empirical Bayes analysis was used to evaluate the posterior probability (pp) that each codon belonged to the site class of positive selection on the foreground branch in the BSM.

### 2.6 Motif and *cis*‐element analyses

All verified SVP proteins were used to search against the Pfam database for additional known domains or motifs with the PfamScan program (https://www.ebi.ac.uk/Tools/pfa/pfamscan/) (Madeira et al., 2019). The promoter sequences of the *SVP* genes were extracted from the genome sequences on the basis of gff3 files by using local Python scripts. The *cis*‐acting elements of putative promoters (1500 bp upstream of the annotated transcripts) were identified using the PlantCARE program (http://bioinformatics.psb.ugent.be/webtools/plantcare/html/) (Lescot et al., 2002).

### 2.7 Sample collection and qRT‐PCR

Five‐year‐old seedlings of *Rosa multiflora* carnea Thory were grew in an outdoor environment. Axillary buds and leaf samples were collected in liquid nitrogen at monthly intervals from 15 July 2018 to 15 June 2019, and stored at −80 °C until RNA extraction. At each axillary bud sampling, a part of the axillary bud was vertically dissected into two equal parts under a stereo microscope (Fluorescent Stereo Microscope Leica M165 FC, Wetzlar, Germany) to determine the development and differentiation period. The differentiation period was determined as described by Liu et al. (2018). Total RNA from *Rosa multiflora* axillary buds and leaves was extracted using the BioTeke Quick RNA Isolation Kit (Cat.RP3301, BioTeke Corporation, Beijing, China); 1 μg of high‐quality total RNA was reverse‐transcribed using the PrimeScript RT Reagent Kit (Cat. RR047A; TaKaRa, Dalian, China), according to the manufacturer's instructions. Fluorescent quantitative primers for *SVP* genes were designed using GenScript (https://www.genscript.com/) and IDT (http://sg.idtdna.com/scitools/Applications/RealTimePCR/) online server. qRT‐PCR analysis was performed using the QuantStudio 6 RealTime PCR System (Thermo Fisher Scientific, California, USA) with SYBR Premix Ex Taq (Tli RNaseH Plus; catalog number: RR420A, TaKaRa), according to the manufacturer's instructions. Three biological repetitions and three technical repetitions were performed for each experiment. *GAPDH* was used as the reference gene (Liu et al., 2018), and the 2^–ΔCT^ method was used to calculate the relative expression of the rose *SVP* genes. The qRT‐PCR primers were listed in Supplementary Table S1.

## 3 RESULTS

### 3.1 *SVP* genes were significantly expanded in Rosaceae (mostly perennial species) when compared with Brassicaceae (annual species)

**FIGURE 1:**
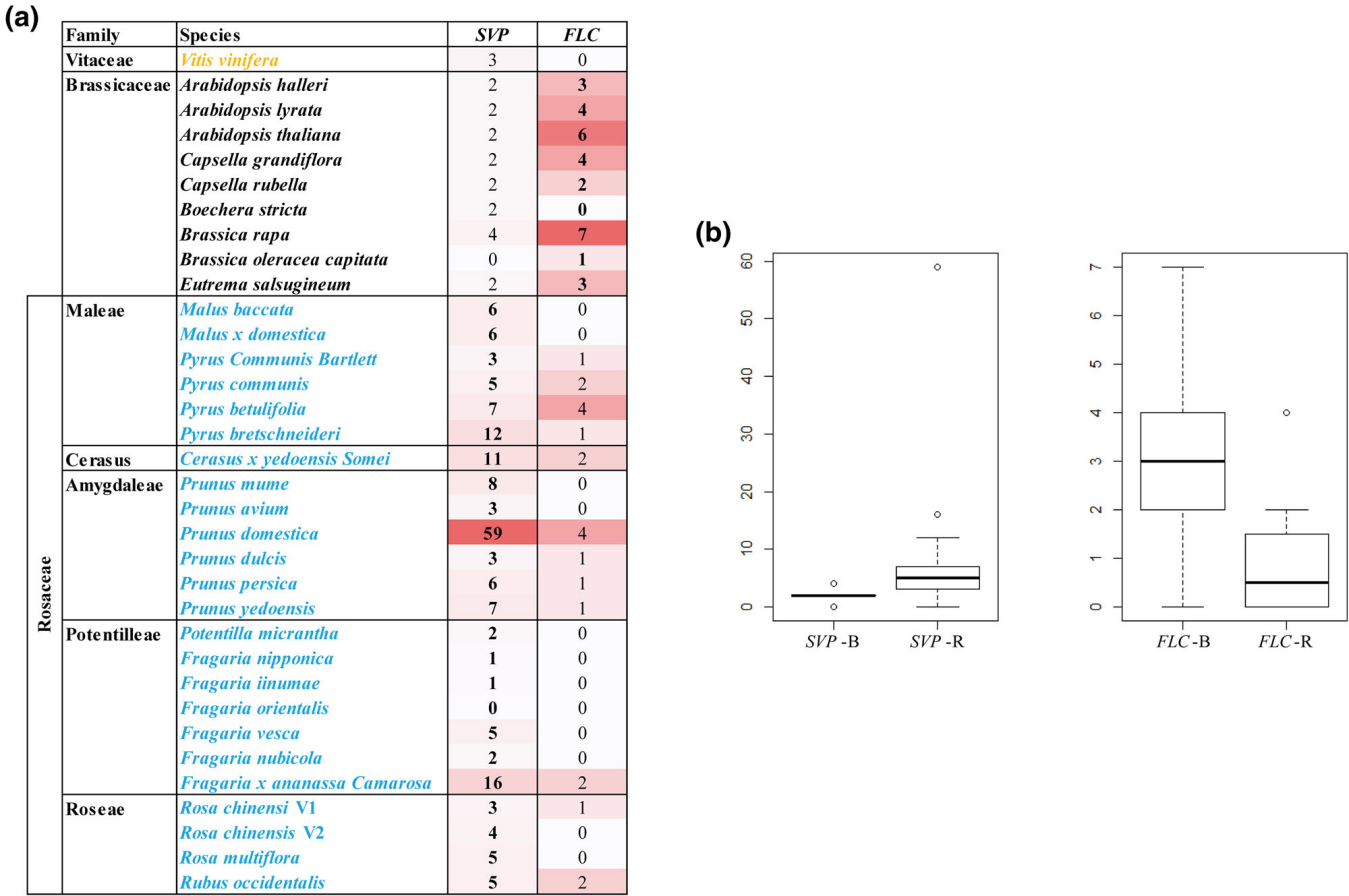
*SVP* and *FLC* genes in Brassicaceae and Rosaceae. (a) List of the sequenced Brassicaceae and Rosaceae species and copy numbers of *SVP* and *FLC* genes identified in our study. (b) Boxplots of the copy numbers of *SVP* and *FLC* genes in Brassicaceae and Rosaceae, respectively. *SVP*‐B and *FLC*‐B denote *SVP* and *FLC* genes, respectively, from Brassicaceae, whereas *SVP*‐R and *FLC*‐R denote *SVP* and *FLC* genes, respectively, from Rosaceae

*FLC* and *SVP* genes have been indicated to be the key regulators of vernalization in annual species, particularly in Brassicaceae (Alexandre & Hennig, 2008; Ream et al., 2012). To gain insights into the evolutionary features of *FLC* and *SVP* genes in Rosaceae (mainly perennials), we performed extensive identification of *FLC* and *SVP* genes in the genomes of Brassicaceae and Rosaceae and generally compared their copy numbers, with *Vitis vinifera* as the outgroup (Figure 1). We identified 18 and 180 *SVP* genes in 9 Brassicaceae species and 24 Rosaceae species, respectively. Moreover, we detected 30 and 22 *FLC* genes in Brassicaceae and Rosaceae, respectively (Supplementary Table S2). The copy number of *SVP* genes in Brassicaceae ranged from 0 to 4 with an average of 2, whereas it ranged from 0 to 59 with an average of 7.5 in Rosaceae (Figure 1a), which was significantly higher than that in Brassicaceae (Figure 1b). Conversely, the copy number of *FLC* genes in Rosaceae ranged from 0 to 4 with an average of 0.9 (Figure 1a), which was significantly lower than that in Brassicaceae (ranging from 0 to 7 with an average of 3.3; Figure 1b). Our results indicated that the *SVP* genes were significantly expanded in Rosaceae, whereas numerous *FLC* genes were lost during the evolution of Rosaceae.

### 3.2 Phylogenetic analysis classified all Rosaceae *SVP* genes into five clades, and SVP2‐R1, SVP2‐R2, and SVP2‐R3 clades were lineage‐specific

**FIGURE 2:**
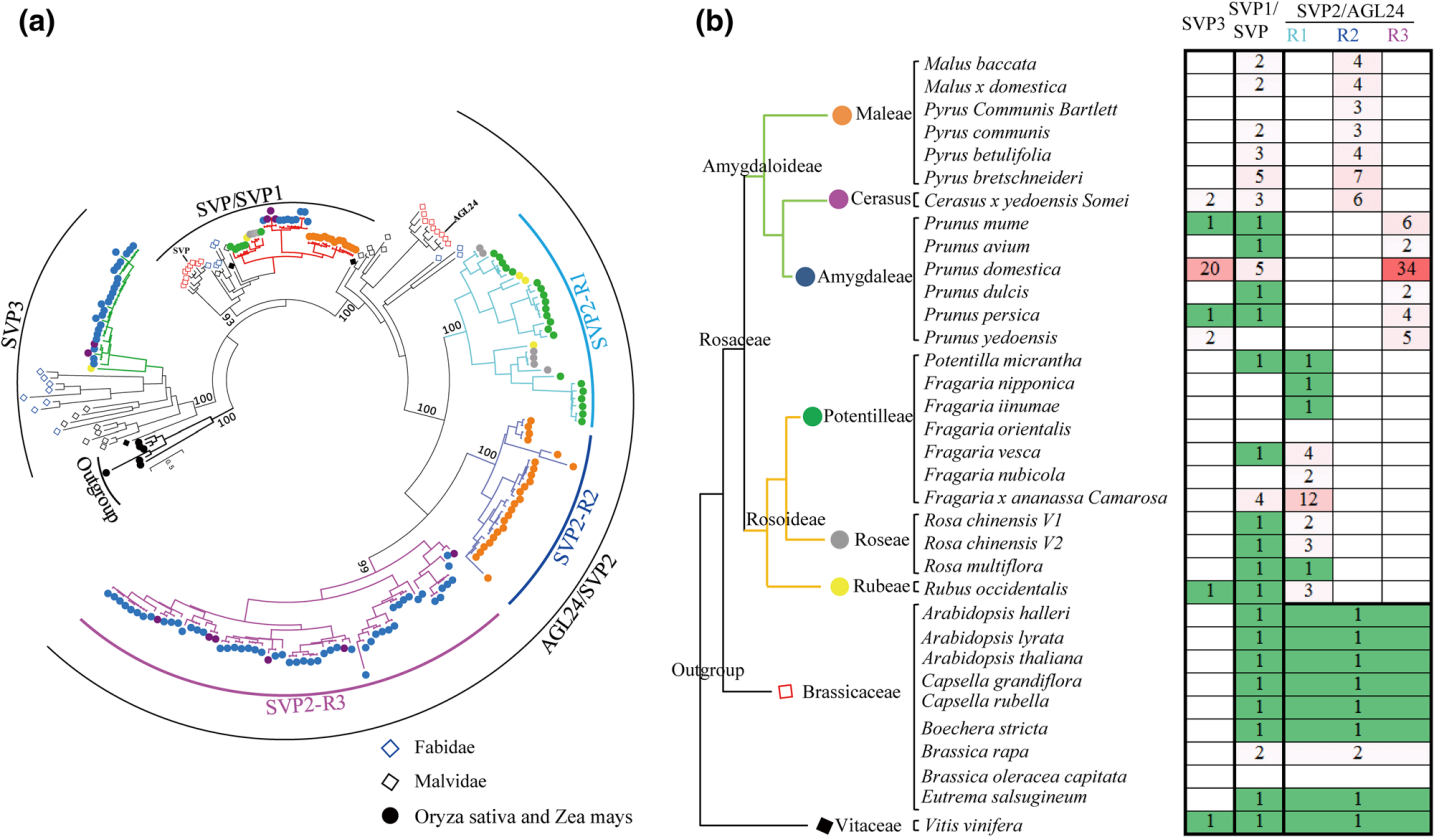
Phylogeny of *SVP* genes in Rosaceae (a) Phylogenetic tree of all *SVP* genes from 24 Rosaceae, 9 Brassicaceae species, 2 other Malvidae species, 3 other Fabidae species, *Vitis vinifera* (basal Rosids) and 2 Poaceae species. Phylogenetic tree was constructed using FastTree and IQTREE software (see Materials and Methods section for details) (Supplementary Figure S4 and S5). After placing SVP homologies from *Oryza sativa* and *Zea mays* as outgroup. The *SVP* genes from Rosaceae were classified into three major clades (SVP/SVP1, AGL24/SVP2 and SVP3) on the basis of their phylogeny, and Rosaceae AGL24/SVP2 were further classified into three subclades, namely, SVP2‐R1 (R1), SVP2‐R2 (R2) and SVP2‐R3 (R3). (b) Phylogeny of the species and their *SVP* copy numbers for each clade. The tree of the lineages was constructed on the basis of a previous study (Xiang et al., 2017)

To gain more insight into the expansion profiles of *SVP* genes in Rosaceae, we constructed a maximum likelihood (ML) tree by using the full‐length sequences of 235 SVP proteins that from the genomes of 24 Rosaceae, 9 Brassicaceae species, 2 other Malvidae species, 3 other Fabidae species, *Vitis vinifera* (basal Rosids) and 2 Poaceae species. When placed SVP homologies from 2 Poaceae (*Oryza sativa* and *Zea mays*) as the outgroup and on the basis of the topology of the phylogenetic tree, Rosaceae SVPs were divided into three major clades, namely, SVP/SVP1, AGL24/SVP2 and SVP3, with bootstrap values > 93 (Figure 2a), and Rosaceae AGL24/SVP2 were further classified into three subclades, namely, SVP2‐R1 (R1), SVP2‐R2 (R2) and SVP2‐R3 (R3). Interestingly, we found that only the SVP/SVP1 and AGL24/SVP2 clade contained *SVP* genes from Brassicaceae, indicating an extensive gene loss of SVP3 clade in Brassicaceae. Conversely, the subclades (R1, R2 and R3) from AGL24/SVP2 were specifically expanded in Rosaceae. Furthermore, to investigate their expansion profiles in detail, we counted the copy numbers of SVP clades along with the phylogeny of 24 Rosaceae, 9 Brassicaceae species and *Vitis vinifera* (basal Rosids) (lineage names were marked with colored circles), and found that R2 and R3 were Amygdaloideae‐specific (R3 was further found to be Amygdaleae‐specific) and R1 was Rosoideae‐specific (Figure 2b). Our results indicate that the R1, R2 and R3 clades originated from AGL24 clade genes and specifically expanded in different lineages of Rosaceae.

### 3.3 Evolution profiles of *SVP* genes in Rosaceae: Lineage‐specific gene duplication events

**FIGURE 3:**
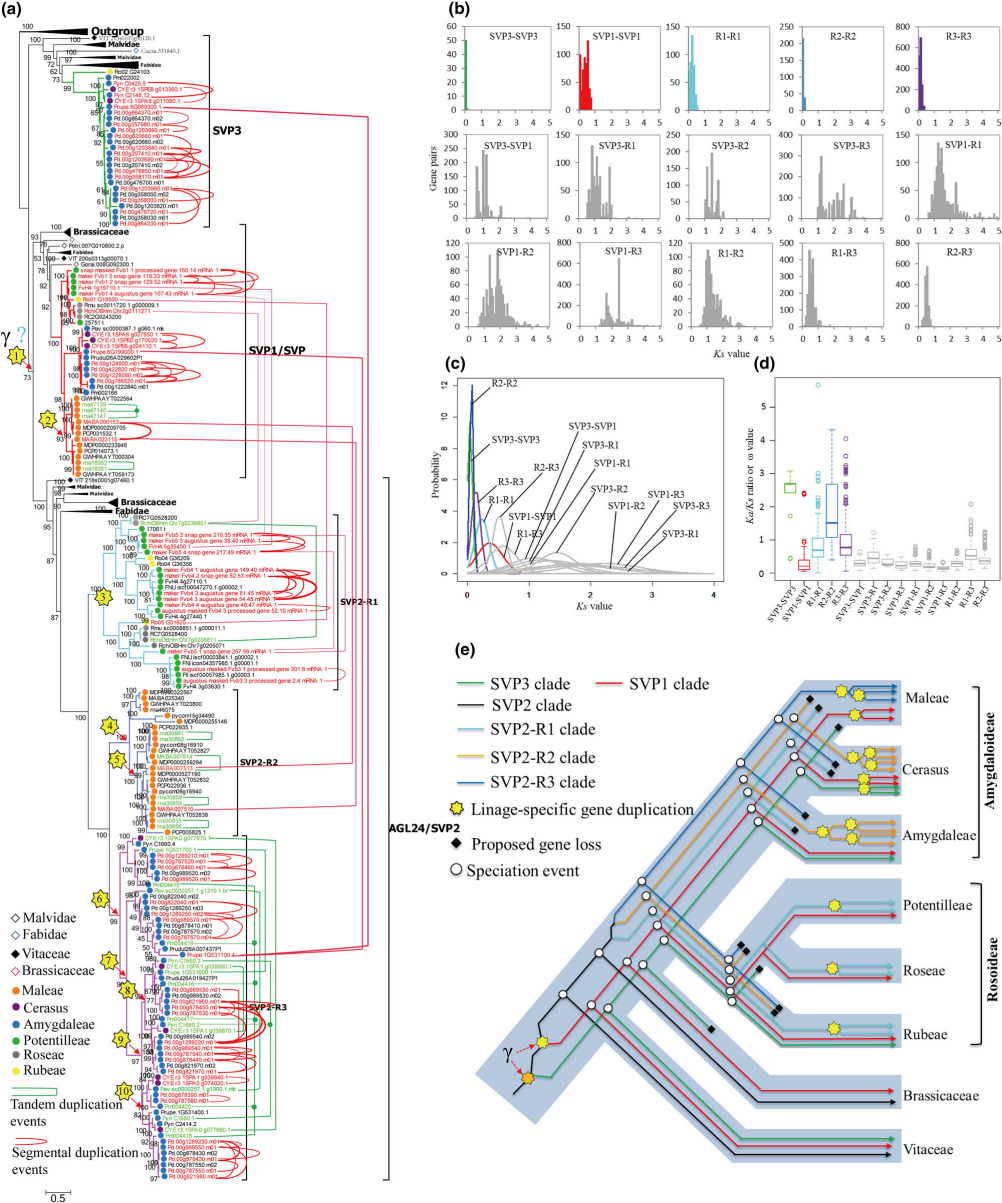
Gene duplication (GD), synteny analysis, and evaluation of nonsynonymous substitutions and synonymous substitutions of *SVP* genes in Rosaceae. (a) The phylogenetic tree of *SVP* genes from 24 Rosaceae, 9 Brassicaceae species, 2 other Malvidae species, 3 other Fabidae species, *Vitis vinifera* (basal Rosids) and 2 Poaceae species (SVP homologies from *Oryza sativa* and *Zea mays* were placed as outgroup) and their lineage‐specific gene duplication events (yellow stars). (b) *Ks* distribution of paralogous *SVP* genes within∖between clades. (c) Normal distributions fit to *Ks* values for compositions of clades that correspond to (b). (d) Boxplot of the *Ka*/*Ks* values within∖between SVP clades. (e) A hypothetical model for the evolution of *SVP* genes in Rosaceae

The gene identification and phylogenetic analysis indicated that *SVP* genes were significantly expanded in Rosaceae in a lineage‐specific manner, indicating potential lineage‐specific gene duplication (GD) events and possible functional divergence as well as adaptive evolution of *SVP* genes in Rosaceae. On the basis of phylogenetic analysis, we detected and proposed 10 GD events for *SVP* genes during the evolution of Rosaceae and named them GD1–GD10 (Figure 3a). Among them, GD1 was found to occur before the evolution of Rosaceae, which contribute to major diversifications of *SVP* genes and may also generate the ancestral clade of R1, R2, and R3 clade genes in Rosaceae. Besides, other GDs were indicated to be lineage‐specific in Rosaceae. For example, GD4–10 were indicated to be Amygdaloideae‐specific, with GD4 being further Maleae‐specific, while GD3 was Rosoideae‐specific. Furthermore, synteny analysis was performed to gain more insights into the expansion profiles of *SVP* genes in Rosaceae. As shown in Figure 3a and Supplementary Table S3, we detected 20 tandem duplication events (green lines) and 83 segmental duplication events within Rosaceae species, that are related to 35 (21%) and 81 (48.5%) out of 167 *SVP* genes, respectively, indicating that both the tandem and segmental duplication events were the major forces contributed to the expansion of *SVP* genes in Rosaceae. Interestingly, most of the segmental duplication events were detected within clades, with only 9 (10.8%) out of 81 found to be between different clades; no segmental duplication events were detected between SVP/SVP1 and SVP3, R1 and R2, R1 and R3, and R2 and R3 clades, suggesting that all 3 SVP clades (R1, R2, and R3) independently originated from their ancestral clade and might be contributed mainly by GD1. In addition, we found that the distribution of tandem duplication events were uneven across the different clades, and most of them were detected in only R2 and R3 clades; this suggested that the evolution profile of the tandem duplications of *SVP* genes was Amygdaloideae‐specific.

To better understand the evolutionary characteristics of *SVP* genes in different clades, synonymous divergence of *SVP* genes was analyzed within/between clades (Figure 3b, c). The results showed that the distribution of *Ks* values for different clades and their combinations varied significantly (Figure 3b), suggesting different evolution courses for different SVP clades in Rosaceae. Moreover, normal distributions that fit pairwise *Ks* values for each combination of SVP clades demonstrated the timing of GDs. Inter‐clade combinations (gray line) were used to estimate the timing of GD events that led to the divergence of the 5 SVP clades (Figure 3c). Among them, the peaks of the *Ks* distributions of SVP3‐SVP1, SVP3‐R1, SVP3‐R2, SVP3‐R3, SVP1‐R1, SVP1‐R2 and SVP1‐R3 were at 1.05, 1.0, 1.55, 1.7, 1.6, 1.65 and 1.65, respectively, which were significantly higher than that of R1‐R2 (peak is 1.0), R1‐R3 (0.95), and R2‐R3 (0.55). This suggested that SVP1 was the ancestral clade and diverged before the divergence of R1, R2, and R3, and the R1 clade further diverged before the divergence of R2 and R3. These results were in consistent with the phylogenetic tree (Figure 3a). Intra‐clade comparisons (SVP1‐SVP1, SVP3‐SVP3, R1‐R1, R2‐R2 and R3‐R3) were used to estimate the timing of the GD events after the divergence of the SVP1, SVP3, R1, R2 and R3 clades. As shown in Figure 3c, the peaks of the *Ks* distributions of SVP1‐SVP1, SVP3‐SVP3, R1‐R1, R2‐R2 and R3‐R3 were observed to be at 0.35, 0.05, 0.3, 0.1 and 0.2, respectively. This result also supported the fact that SVP1 was the ancestral clade and the timing order of the divergence for the other clades.

The *Ka*/*Ks* ratio was determined to assess the selective pressure for all the SVP clade combinations (Figure 3d). Most of the gene pairs (99.2%) were found to have *Ka*/*Ks* ratios less than 1 (Supplementary Table S4), indicating that most of the *SVP* genes underwent purifying selection during their evolution in Rosaceae. However, the strengths of the purifying selections were uneven within or between different clades. Generally, the *Ka*/*Ks* ratios within SVP3, R1, R2 and R3 clades were significantly higher than those in the other clades and their combinations (Figure 3d), suggesting that the purifying selection strengths of SVP3, R1, R2 and R3 clades were less than that of other clades during their evolution. Out of the selected gene pairs, 65 gene pairs had *Ka*/*Ks* ratios greater than 1 (Supplementary Table S4), indicating that these genes were driven by positive selection. Interestingly, a high proportion of the positive selected gene pairs were also found to be from R1, R2 and R3 clades, suggesting their prominent roles relevant to species adaptation to bud dormancy in Rosaceae.

Finally, on the basis of results and phylogeny of Rosaceae species in a previous study (Xiang et al., 2017), we proposed a model for GD and extensive gene loss for *SVP* genes during their evolution in Rosaceae. Thus, we have provided comprehensive evolutionary profiles for the *SVP* genes in Rosaceae.

### 3.4 Positive selection analysis and sequence conservation or variation among SVP clades in Rosaceae

**FIGURE 4:**
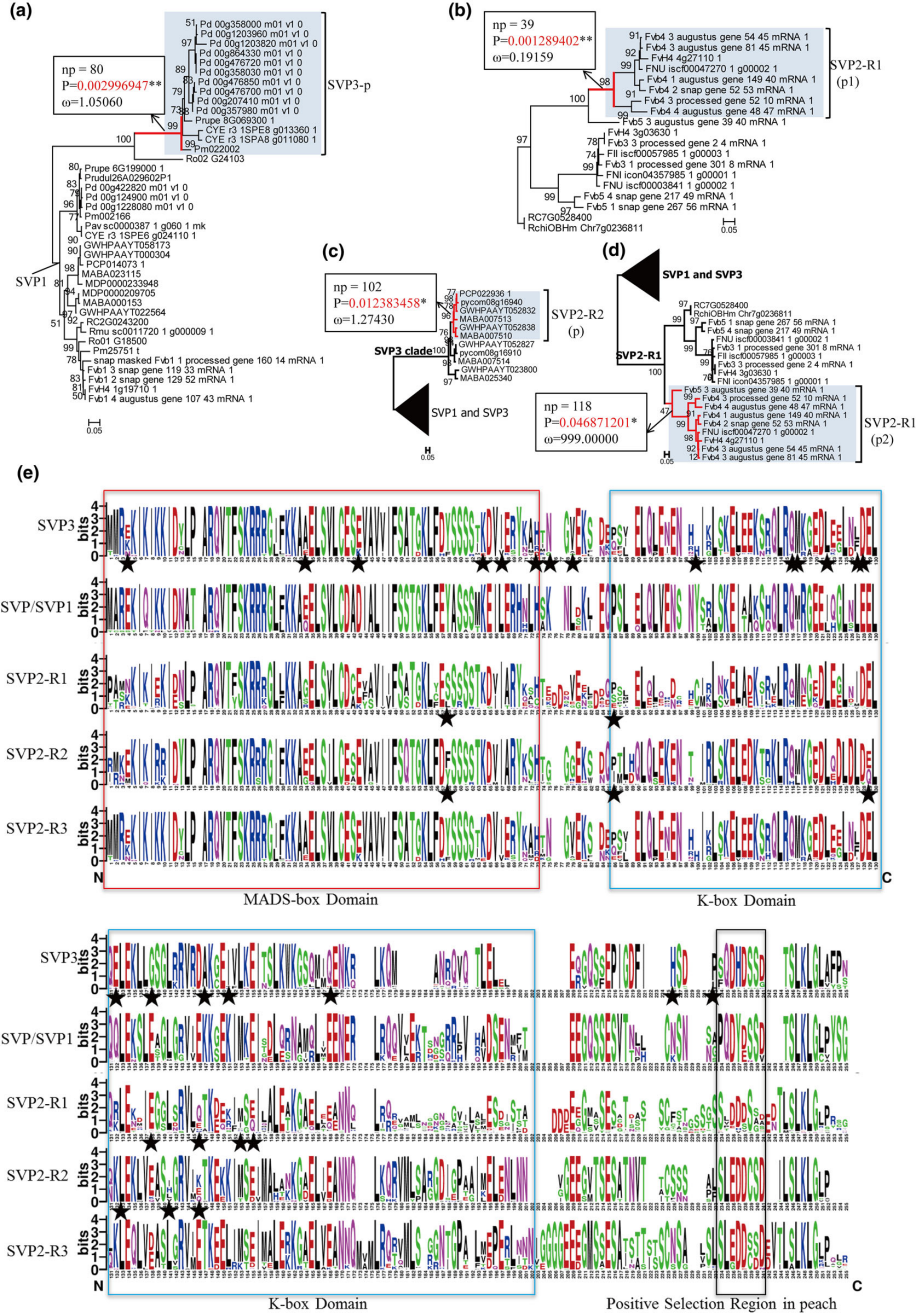
Positive selections and sequence logos of the 5 SVP clade genes in Rosaceae. (a and b) Positive selections detected with the branch model within SVP3 and R1 clades, respectively. (c and d) Positive selections detected with the branch model within R1 and R2 clades when the SVP/SVP1 and SVP3 clade was included for comparison. (E) Sequence logos and positive selection sites (detected with branch site models) of the 5 SVP clade genes. Sequence alignments of SVP proteins were generated using MAFFT v7.037b software (Katoh & Standley, 2013) with the same parameters as those in Figure 2a and Figure 3a. The large unaligned regions were removed manually by using MEGA7 software (https://www.megasoftware.net/home) (Kumar et al., 2016). Positive selection sites are marked with black stars. MADS‐box and K‐box domains and potential positive selection sites that were predicted specifically for the 6 peach *DAM* genes in a previous study (Jimenez et al., 2009) are marked with boxes

The evolution analysis suggested that *SVP* genes might have undergone evolutionary selection in Rosaceae. To gain more insight into their selection constraints during evolution, we investigated selection models for different clades of *SVP* genes by using EasyCodeML software (see Materials and Methods section for details). Using the BM, our result showed a significant difference between the null model and two‐ratio model (P = .049662201) for R1, R2, and R3 clades (foreground) when the SVP1 and SVP3 clade was set as the background (Table 1), and the ω values (dN/dS) were 0.13721, 0.13839, and 0.16571, respectively. Conversely, when R1, R2, and R3 clades were set as the background, the ω value of the SVP/SVP1 and SVP3 clade (foreground) was found to be 2.14064 (ω > 1) with no significant P value (Table 2). These results suggest that *SVP* genes might have undergone adaptive selection during evolution in Rosaceae. In addition, the selection models within and between different clades were extensively investigated and BSMs were used to test the possible positively selected sites simultaneously. Four sub‐clades from SVP3, R1, and R2 clades were found to have undergone adaptive selection with statistically significant P values (Figure 4a–d); with the BSMs, 23, 6, and 6 potential positive selection sites were found in SVP3, R1, and R2 clades, respectively, with statistically significant P values (Supplementary Table S5; Supplementary Figure S6). No positive selection sites were detected in the SVP/SVP1 clade.

**TABLE 1:** Likelihood ratio test for the branch model in the R1, R2, and R3 clades when SVP3 and SVP1 clade was set as the background branch in Rosaceae

			Estimate of parameter ω			
Model	np	Ln L	SVP3‐R1	SVP2‐R2	SVP2‐R3	P‐value
Two ratio Model 2	270	−10424.347620	0.13721	0.13839	0.16571	.049662201^*^
Model 0	267	−10428.262546		0.31159		

^*^ Significant at the .05 probability level.

**TABLE 2:** Likelihood ratio test for the branch model in the SVP3 and SVP1 clade when R1, R2, and R3 were set as the background branches in Rosaceae

Model	np	Ln L	Estimate of parameter ω	P‐value
Two ratio Model 2	268	−10429.743180	2.14064	.085281039
Model 0	267	−10428.262546	0.31159	

To observe the conserved and variant features of Rosaceae SVP sequences as well as distribution of the potential positive selection sites in different clades, the sequence logos were generated to visualize their components for each clade. As shown in Figure 4e, although the protein structures of *SVP* genes, including MADS‐box domain, K‐box domain, and potential positive selection site region, were highly conserved in different clades, some of the amino acid residues were found to be highly divergent within and across different clades and clade‐specific. For example, in the MADS‐box domain, the amino acid residues (R) at site 3 were highly conserved in the SVP3, SVP/SVP1 and R3 clades, whereas they were completely different in the R1 and R2 clades; the amino acid residues at site 14 (L), 15 (P), 46 (V), 65 (E), and 70 (Y) were highly conserved in R1, R2, and R3, but completely different in SVP3 and SVP/SVP1. Our results suggest that these sites may be related to variations in the binding properties of the MADS‐box domain for *SVP* genes in different clades or different species lineages. Moreover, high site variations were found in the K‐box domains, suggesting the functional diversification of *SVP* genes in Rosaceae. In addition, higher proportions of potential positive selection sites were found in the K‐box domains (Figure 4e, black stars), suggesting their important roles in the functional diversification of *SVP* genes during evolution in Rosaceae. Interestingly, the potential positive selection site region (black box) predicted for the 6 peach *DAM* genes (belonging to R3 clade) during evolution in a previous study was highly conserved within the R2 clade, whereas it varied in the other clades, especially R1 (Figure 4e). This finding suggests that this region is very important for *SVP* genes in bud dormancy, implying that the biological functions of R1 clade genes may diverge from those of the other clades.

### 3.5 Promoter *cis*‐acting element analysis of *SVP* genes

**FIGURE 5:**
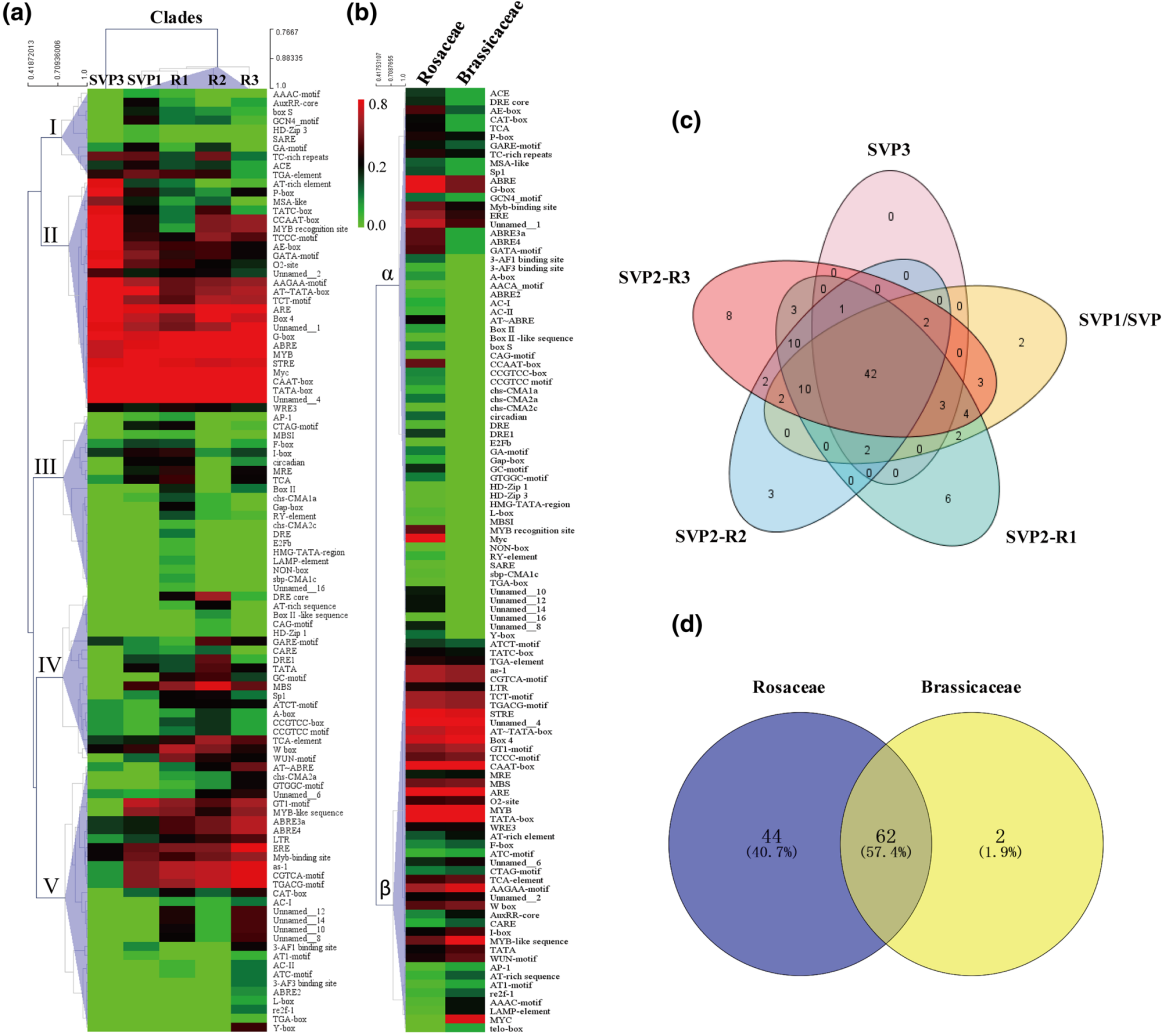
Comparative analyses of the promoter *cis*‐acting elements between different clades of *SVP* genes from Rosaceae and Brassicaceae. (a) The number of *cis*‐acting elements per gene between different clades of *SVP* genes in Rosaceae. (b) The number of *cis*‐acting elements per gene of *SVP* genes between Rosaceae and Brassicaceae. (c) Venn diagrams of the distribution of promoter *cis*‐acting elements in different clades of Rosaceae *SVP* genes. (d) Venn diagrams of the distribution of promoter *cis*‐acting elements of *SVP* genes between Rosaceae and Brassicaceae

To understand the mechanism of transcriptional control, we identified the *cis*‐acting elements of 1500 bp upstream promoter regions of *SVP* genes by using the PlantCARE program (see Materials and Methods section for details) and compared their copy numbers between different clades and between Rosaceae and Brassicaceae. As shown in Figure 5 and Supplementary Table S6, we identified 106 and 64 *cis*‐acting elements in the promoter regions of *SVP* genes from Rosaceae and Brassicaceae, respectively. To compare the *cis*‐acting elements between different clades and lineages, the number of *cis*‐acting elements per gene was calculated and presented as clustered heatmaps. Five clusters (I–V) were observed for different clades of *SVP* genes from Rosaceae. Among them, the *cis*‐acting elements of clusters II were highly presented in the promoter regions of all clades of SVP genes, especially in that of SVP3 clade genes, indicating that those *cis*‐acting elements were highly conserved within Rosaceae SVP genes. By contrast, the number of *cis*‐acting elements per gene in clusters I, III, IV and V were relatively higher in different clade and showed a preference for clade specification, suggesting their potential roles that contributed to functional diversifications of SVP genes in Rosaceae (Figure 5a). Furthermore, 2 clusters, α and β, were detected when Rosaceae and Brassicaceae were compared; the presence of cluster α was greater in Rosaceae and that of cluster β was relatively greater in Brassicaceae, suggesting the diversification of regulatory functions of *SVP* genes between Rosaceae and Brassicaceae. Finally, the Venn diagrams showed 2 (HD‐Zip 3 and SARE), 6 (3‐AF3 binding site, ABRE2, L‐box, re2f‐1, TGA‐box, and Y‐box), 3 (Box II ‐like sequence, CAG‐motif, and HD‐Zip 1), and 8 (chs‐CMA2c, DRE, E2Fb, HMG‐TATA‐region, LAMP‐element, NON‐box, sbp‐CMA1c, and Unnamed__16) promoter *cis*‐acting elements of *SVP* genes of SVP/SVP1, R1, R2, and R3 clades, respectively, suggesting their specific roles in the regulatory mechanisms in different clades of Rosaceae (Figure 5c; Supplementary Table S6). Interestingly, the large‐scale comparison of Rosaceae and Brassicaceae showed that 44 (40.7%) *cis*‐acting elements were specific to Rosaceae, whereas only 2 *cis*‐acting elements were specific to Brassicaceae (Figure 5d; Supplementary Table S6); this suggests the dramatic innovation of regulatory mechanisms and biological functions of *SVP* genes in Rosaceae.

### 3.6 Seasonal expression patterns of *SVP* genes in *Rosa multiflora*

**FIGURE 6:**
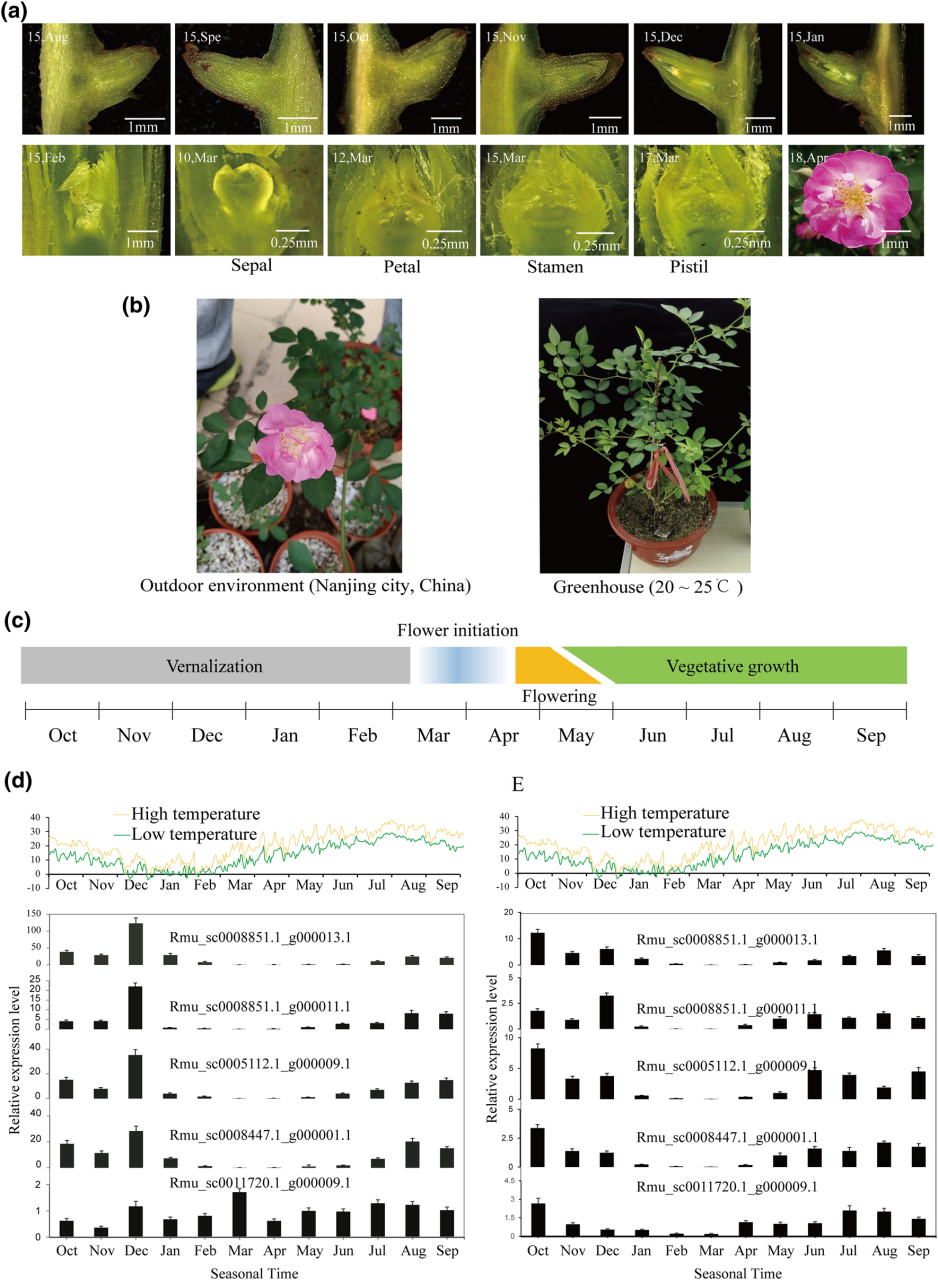
Vernalization of seasonal flowering rose and expression patterns of *RmSVP* genes during the season. (a) The dissected structures of rose buds and flowers. (b) Rose plants maintained under controlled greenhouse and outdoor conditions. (c) Schematic diagram of rose seasonal flowering. (d and e) Relative expression levels of *RmSVP* genes in leaves and bud tissues during the season, determined with qRT‐PCR

In Rosaceae, most of the species, including strawberry, raspberry, and fruit trees that belong to the genera *Malus*, *Pyrus*, and *Prunus*, exhibit flower initiation from late summer to autumn, but flowering occurs in spring. In winter (between autumn and spring), the buds enter a state of dormancy, known as bud dormancy (Falavigna et al., 2019). However, in roses, the process of flower initiation and flowering could occur in the same season (spring), suggesting different regulatory mechanisms for seasonal flowering in rose (Foucher et al., 2008; Kurokura et al., 2013). We hypothesized that winter cold for rose floral transition is vernalization instead of leading to bud dormancy. To test this hypothesis, the dissected structures of the buds were observed during the annual growth cycles of *Rosa multiflora* (a seasonal flowering rose) by using a stereo microscope (Figure 6a). Our results showed that flower initiation in *Rosa multiflora* occurred in spring (early March to April) after the winter cold, and the flowering occurred immediately after flower initiation. Moreover, to test whether the winter cold is necessary for rose flowering, 3‐year‐old seedlings of *Rosa multiflora* were grown in a greenhouse with the environmental temperature no less than 20 °C, with some seedlings grown outdoors as the control. Our results showed that the seedlings in the greenhouse did not flower at all but showed vegetative growth, whereas the outdoor seedlings maintained seasonal flowering (Figure 6b); this suggests that winter cold is necessary for rose flower initiation. On the basis of these results, we proposed a schematic diagram for seasonal flowering of rose (Figure 6c).

To gain insights into the biological roles of rose *SVP* genes in this process, we determined the relative expression levels of all 5 rose *SVP* genes in both leaves and buds during an entire growth cycle. As shown in Figure 6d and e, 4 of the rose *RmSVP* genes, *Rmu_sc0008851.1_g000013.1*, *Rmu_sc0008851.1_g000011.1*, *Rmu_sc0005112.1_g000009.1*, and *Rmu_sc0008447.1_g000001.1*, had very similar patterns of gene expression in the leaves during the growth cycle. Their expressions were significantly upregulated when the environmental temperature first went to subzero (normally in December) and gradually decreased to a very low level thereafter and maintained until the end of the flowering period (late April to May). Similar expression patterns of the 4 *RmSVP* genes were also found in the buds during the year, in which all the *RmSVP* genes, except *Rmu_sc0008851.1_g000011.1*, were not upregulated in December, and the period of prolonged low expression level was shorter than that in the leaves. Our results indicate that these *SVP* genes are suppressor factors in the process of rose seasonal flowering and may be functionally redundant. Interestingly, we found that the expression pattern of *Rmu_sc0011720.1_g000009.1* was completely different in the leaves and bud tissues, suggesting a functional divergence of *RmSVP* genes.

## 4 DISCUSSION

Bud dormancy is defined as an adaptive process that helps perennial plants to overcome the harsh environmental conditions during winter in temperate climates (Falavigna et al., 2019). The bud dormancy cycle has gained significant attention in the last 2 decades, especially because of the agronomical disorders caused by global warming (Falavigna et al., 2019; Yu, Luedeling, & Xu, 2010). Currently, *SVP* clade genes (also called *DAM* genes), which are homologous to the *Arabidopsis thaliana* floral regulators *SHORT VEGETATIVE PHASE* (*SVP*) and *AGAMOUS*‐*LIKE 24* (*AGL24*), have been shown to play prominent roles in the dormancy cycle of numerous fruit species (Falavigna et al., 2019), especially apple (Wu et al., 2017), pear (Saito et al., 2015), peach (Bielenberg et al., 2008; Jimenez et al., 2010), *Prunus mume* (Zhao et al., 2018), sweet cherry (Rothkegel et al., 2017) and *Prunus mume* (Sasaki et al., 2011). However, the evolutionary history and functional diversification of *SVP* genes in Rosaceae remain unclear. With the large‐scale release of whole‐genome sequences in Rosaceae, we performed genome‐wide identification and evolutionary analysis of *SVP* genes in Rosaceae and presented comprehensive evolutionary features and functional diversification of *SVP* genes during evolution. Our results facilitated the understanding of *SVP* genes with respect to their evolution and function diversifications in Rosaceae.

Crucial differences exist between vernalization in annual plants (like *Arabidopsis thaliana*) and bud dormancy release in perennial plants (Rohde & Bhalerao, 2007; Wellensiek, 1962, 1964). Both processes have large similarities and associations (Chouard, 1960; Rohde & Bhalerao, 2007). Particularly, a certain period of chilling is required to accomplish the reproductive phase, and both of them are regulated by *SVP* genes (Rohde & Bhalerao, 2007). Furthermore, both processes are regulated by histone modifications and DNA methylation, day length, and plant hormones in a similar manner (Falavigna et al., 2019). These results suggested that the processes of bud dormancy and vernalization might have the same origin but different evolutionary courses in different plant lineages. The FLC‐SVP model has been indicated to be the key regulator of vernalization in Brassicaceae (annual plants) (Kurokura et al., 2013; Li et al., 2008; Mateos et al., 2015). However, the evolution profiles and functional diversification of this model in other plant lineages, especially in Rosaceae (mostly perennial plants), are not yet fully understood. In our study, extensive genome‐wide identification of *FLC* and *SVP* genes was first performed in Brassicaceae (9 species) and Rosaceae (24 species). The average copy number of *SVP* genes was 3.75‐fold higher in Rosaceae than in Brassicaceae, whereas the average copy number of *FLC* genes was 3.67‐fold lower in Rosaceae than in Brassicaceae. No *FLC* genes were identified in most of the Rosaceae species (Figure 1), indicating that *SVP* genes were significantly expanded in Rosaceae and *FLC* genes were extensively lost in Rosaceae during evolution. On the basis of increasing evidence of the importance of *SVP* genes in the dormancy cycle of Rosaceae (Alexandre & Hennig, 2008; Falavigna et al., 2019; Kurokura et al., 2013; Rohde & Bhalerao, 2007) and nearly no evidence for *FLC* genes in Rosaceae, we suggest that the biological functions of *SVP* genes may have been evolutionarily enhanced in Rosaceae. Interestingly, the copy numbers of *SVP* genes were found to be relatively lower in diploid *Fragaria* species than in other Rosaceae species; no *SVP* and *FLC* genes were detected in *Fragaria orientalis*, suggesting that *Fragaria* may have specific mechanisms for the seasonal growth cycles and reproductive success that differ from those of other species in Rosaceae.

Our phylogenetic analysis indicated that *SVP* genes in Rosaceae could be divided into three major clades (SVP/SVP1, AGL24/SVP2 and SVP3) according to the topology of the phylogenetic trees (Figure 2a and Figure 3a), which are in consistent with the result from the previous study (Liu et al., 2018). In addition, in order to gain more insights into the detailed evolution of *SVP* genes in Rosaceae, we further classified Rosaceae AGL24/SVP2 clade into three subclades, namely, SVP2‐R1 (R1), SVP2‐R2 (R2) and SVP2‐R3 (R3). We performed a genome‐wide analysis of *SVP* genes in 24 Rosaceae species, which cover 2 major lineages (Rosoideae and Amygdaloideae) of Rosaceae, and found that SVP1 clade genes were present in almost all the species as well as in the outgroup species, including Brassicaceae and *Vitis vinifera* (basal Rosids), suggesting that SVP/SVP1 clade genes were highly conserved in plants (Kaufmann, Melzer, & Theissen, 2005). In contrast, R1–R3 clade genes were detected in specific lineages of Rosaceae (Figure 2b), indicating that these clades were specifically expanded in Rosaceae. These results are in consistent with those of a previous study showing that SVP/SVP1 and AGL24/SVP2 clades are highly conserved and present in nearly all Eudicots and SVP3 were suffered extensive gene loss during evolution and with no genes present in annual plants such as Brassicaceae (Liu et al., 2018b) and also in consistent with the results of a phylogenetic analysis of *SVP* genes in another previous study (Falavigna et al., 2019). Collectively, our results also inferred the innovations or diversification of *SVP* gene functions and their corresponding regulatory mechanisms in Rosaceae.

GD was regarded as the main driving force for acquiring new genes and creating genetic novelty in organisms (Liu et al., 2018; Prince & Pickett, 2002; Taylor & Raes, 2004b). The MADS‐box gene family, which plays key roles in the evolution of plant morphology, originated from GD and dramatically expanded in angiosperms (Airoldi & Davies, 2012; Becker, Winter, Meyer, Saedler, & Theissen, 2000; Gramzow & Theissen, 2013, 2015). The expansion and function divergences of the MADS‐box genes were tightly linked to the evolution of plant body plans and life strategies (Chen, Zhang, Liu, & Zhang, 2017; Gramzow & Theissen, 2010; Liu et al., 2018). In our study, 10 lineage‐specific GD events (GD1–10) were detected and proposed for *SVP* genes in Rosaceae on the basis of phylogenetic analysis (Figure 3a). GD1 contributed to the divergence of *SVP* genes in Brassicaceae and their homologs in *Vitis vinifera*, suggesting that the GDs of *SVP* genes occurred before the divergence of Rosaceae, which may have occurred even before the divergence of Rosids. This result was consistent with the hypothesis of a previous study (Liu et al., 2018b), implying that GD1 in our study may also be regarded as an ancient WGT (γ) event that occurred ∼140 million years ago after the monocot–dicot split and before the separation of the asterid and rosid clades (Bowers, Chapman, Rong, & Paterson, 2003; Jaillon et al., 2007; Liu et al., 2018b). Besides, 9 GD events (GD2–10) were newly found to be Rosaceae‐specific in our study; GD4–10 were Amygdaloideae‐specific (GD4 and 5 was further detected to be Maleae‐specific) and GD3 was Rosoideae‐specific, suggesting their important roles in contributing to the expansion of the *SVP* gene family in Rosaceae. In combination with the collinearities between the different clades (Figure 3a), we suggest that GD1 may not only contributed to major diversifications of *SVP* genes but also generated the ancestral clade that lead to the origin of R1, R2, and R3 clade in Rosaceae. Furthermore, each of the R1–R3 clades was detected in only specific lineages of Rosaceae; therefore, we specified that both GDs and extensive gene loss simultaneously existed for *SVP* genes in Rosaceae during their evolution (Figure 3d).

Tandem duplications have been indicated to be one of the evolutionary features of *SVP* genes in some perennial species (Bielenberg et al., 2008; Falavigna et al., 2019), peach (Bielenberg et al., 2008), and other species such as *Prunus mume* and *Populus trichocarpa* (Porto et al., 2016; Sasaki et al., 2011), suggesting their important roles in the expansion of the *SVP* gene family. In our study, we found that the expansion of *SVP* genes in Rosaceae was widely attributable to tandem and segmental duplications (Figure 3a): 21% (35 genes) and 48.5% (81) of 167 *SVP* genes, respectively. Interestingly, we found that the tandem duplication events were unevenly distributed across the different clades of *SVP* genes. Most of these events were detected in the R2 and R3 clades. This result suggested that the evolutionary profile of tandem duplications for *SVP* genes in Rosaceae might be Amygdaloideae‐specific.

Bud dormancy is described as an adaptive process for perennial plants in temperate climates (Falavigna et al., 2019). The expansion of *SVP* genes, as the key regulatory genes of this process, in Rosaceae may be regarded as adaptive evolution. In our study, 99.2% gene pairs had *Ka*/*Ks* ratios less than 1, indicating that most of the *SVP* genes underwent purifying selection during their evolution (Figure 3d). This result is consistent with that of a previous study on the selection strengths of peach *DAM* genes (Jimenez et al., 2009), in which strong purifying selection that constrains functional divergence among the peach *DAM* genes was proposed (Jimenez et al., 2009). Nevertheless, it is worth mentioning that the purifying selection strengths of SVP3, R1, R1 and R3 clades were less than that in other clades during their evolution (Figure 3d), suggesting that those clades contributed more to their functional diversity or plant adaptation in Rosaceae. In addition, 65 gene pairs (*Ka*/*Ks *> 1) were driven by positive selection (Supplementary Table S4), signifying their important roles in species adaptation to bud dormancy in Rosaceae. Furthermore, the EasyCodeML software (Gao et al., 2019) was used to evaluate the positive selection and verify the results. On the basis of BM, our results indicated that R1, R1 and R3 clades underwent strong purifying selection when the SVP1 and SVP3 clade was set as the background branch (Table 1), whereas the SVP1 and SVP3 clade underwent positive selection when R1, R1 and R3 clades were set as the background (Table 2). This further confirmed that *SVP* genes with respect to bud dormancy were constrained by purifying selection during evolution. Using BSM, we still found some positive selection sites within the sub‐clade (Figure 4e; Supplementary Figure S6; Supplementary Table S5), suggesting their important roles in the functional diversification of Rosaceae *SVP* genes. Our results provide detailed profiles of the selection pressure of *SVP* genes in Rosaceae during evolution.

The evolutionary analyses showed that the *SVP* genes were expanded in a lineage‐specific manner in Rosaceae, indicating innovations or diversification of their biological functions. Sixty‐two *cis*‐acting elements (cluster α) were found to be highly present in the 1500 bp upstream promoter regions of *SVP* genes in Rosaceae (perennial plants) when compared with the annual plants in Brassicaceae; 44 *cis*‐acting elements that accounted for 40.7% of the total were Rosaceae‐specific, indicating differences in their regulatory mechanisms in Rosaceae and Brassicaceae (Figure 5d). Interestingly, some *cis*‐acting elements, such as ABRE, ABRE2, ABRE3a, and ABRE4, play critical roles in ABA signaling, and DRE, DRE core, and DRE1, which are important for the response to low temperature and dehydration, were highly expressed in the promoter regions of the *SVP* genes from Rosaceae (Figure 5b). These results were consistent with those of biological pathways in the control of bud dormancy in temperate fruit tree species mediated by *DAM* and *SVP*‐like genes (Falavigna et al., 2019). In addition, the preferences of the *cis*‐acting elements were highly varied between the different clades of *SVP* genes in Rosaceae (Figure 5a, c), suggesting their functional diversification in different lineages.

Currently, numerous studies have been performed for the functional characterization of *SVP* genes in Rosaceae (Falavigna et al., 2019; Kurokura et al., 2013; Rohde & Bhalerao, 2007), and the *SVP*/*DAM* genes have been shown to present seasonal expression patterns along the year cycle, even among different species (Falavigna et al., 2019). However, almost all studies mainly focused on the species from Amygdaloideae (Potter et al., 2007), which belong to specific clades (R2 and R3) of *SVP* genes in our study, and 3 seasonal expression patterns have been proposed for these *SVP* genes (Falavigna et al., 2019). The expression profiles and biological functions of *SVP* genes (referred to as R1 clade genes in our study) from Rosoideae, which is another main subfamily of Rosaceae (Potter et al., 2007), were still unclear. In our study, we dissected the bud structures of *Rosa multiflora* (a seasonal flowering rose) for a cycle and found that the processes of flower initiation and flowering could occur in the same season (Figure 6a). This is different from the species from Amygdaloideae, in which flower initiation and flowering occur in separate growing seasons (Kurokura et al., 2013). In addition, our results showed that winter cold determined the cell divisions and was necessary for flower initiation in *Rosa multiflora* (Figure 6b), which is obviously different from the effects of winter cold on the release of bud dormancy in Amygdaloideae (Rohde & Bhalerao, 2007). Therefore, on the basis of the definitions of and crucial differences between vernalization and release from bud dormancy (Rohde & Bhalerao, 2007), we suggested that the flowering process of *Rosa multiflora* during winter was similar to vernalization and intrinsically different from bud dormancy. Our results are consistent to those of a previous study (Foucher et al., 2008). Furthermore, our results suggest the different expression patterns and regulatory mechanisms of *SVP* genes in *Rosa multiflora*. Indeed, the expression patterns of *RmSVP* genes were found to be different from those in the previous study (Falavigna et al., 2019), but very similar to the expression patterns of *FLC* genes in *Arabidopsis* (Whittaker & Dean, 2017) and *KSN* genes in rose (Randoux et al., 2014). Thus, our results provide novel insights into the functional diversification of *SVP* genes with respect to their roles in processes other than bud dormancy.

In conclusion, we performed a comprehensive evolutionary analysis of *SVP* genes in Rosaceae and revealed that *SVP* genes were significantly expanded in Rosaceae in a lineage‐specific manner that was widely attributable to lineage‐specific GD events. The synteny analysis indicated that the expansion of Rosaceae *SVP* genes was widely attributable to tandem and segmental duplications. Interestingly, tandem duplications were found to be Amygdaloideae‐specific in Rosaceae. The selection pressure and *cis*‐acting elements analyses suggested the large functional innovations and diversification of *SVP* genes in different lineages of Rosaceae. The different growth cycle and novel expression patterns of *SVP* genes in *Rosa multiflora* provided new insights into the functional diversification of *SVP* genes in terms of their roles in processes other than bud dormancy. Our analysis will facilitate further research on *SVP* genes with respect to their evolutionary history and biological functions in Rosaceae.

## AUTHOR CONTRIBUTIONS

CQW and JYL conceived and designed the original research plans; JYL performed the data analysis and wrote the manuscript; RM, HC, SLW, HJY and AJ helped with experimental works and data analysis; CQW and AJ revised the manuscript. All authors reviewed and approved the final manuscript.

## CONFLICT OF INTEREST

The authors declare that they have no conflicts of interest.

## Supporting information

SUPPLEMENTARY MATERIAL

SUPPLEMENTARY MATERIAL

SUPPLEMENTARY MATERIAL

SUPPLEMENTARY MATERIAL

SUPPLEMENTARY MATERIAL

SUPPLEMENTARY MATERIAL

SUPPLEMENTARY MATERIAL

SUPPLEMENTARY MATERIAL

SUPPLEMENTARY MATERIAL

SUPPLEMENTARY MATERIAL

SUPPLEMENTARY MATERIAL

SUPPLEMENTARY MATERIAL
