# Supplementary material for: Evolution of SHORT VEGETATIVE PHASE (SVP) genes in Rosaceae: Implications of lineage‐specific gene duplication events and function diversifications with respect to their roles in processes other than bud dormancy
Source: Plant Genome. 2020 Sep 17;13(3):e20053. doi: 10.1002/tpg2.20053 (PMC12807081; doi:10.1002/tpg2.20053)
Supplement: Supplementary file 1 [file TPG2-13-e20053-s002.pdf]

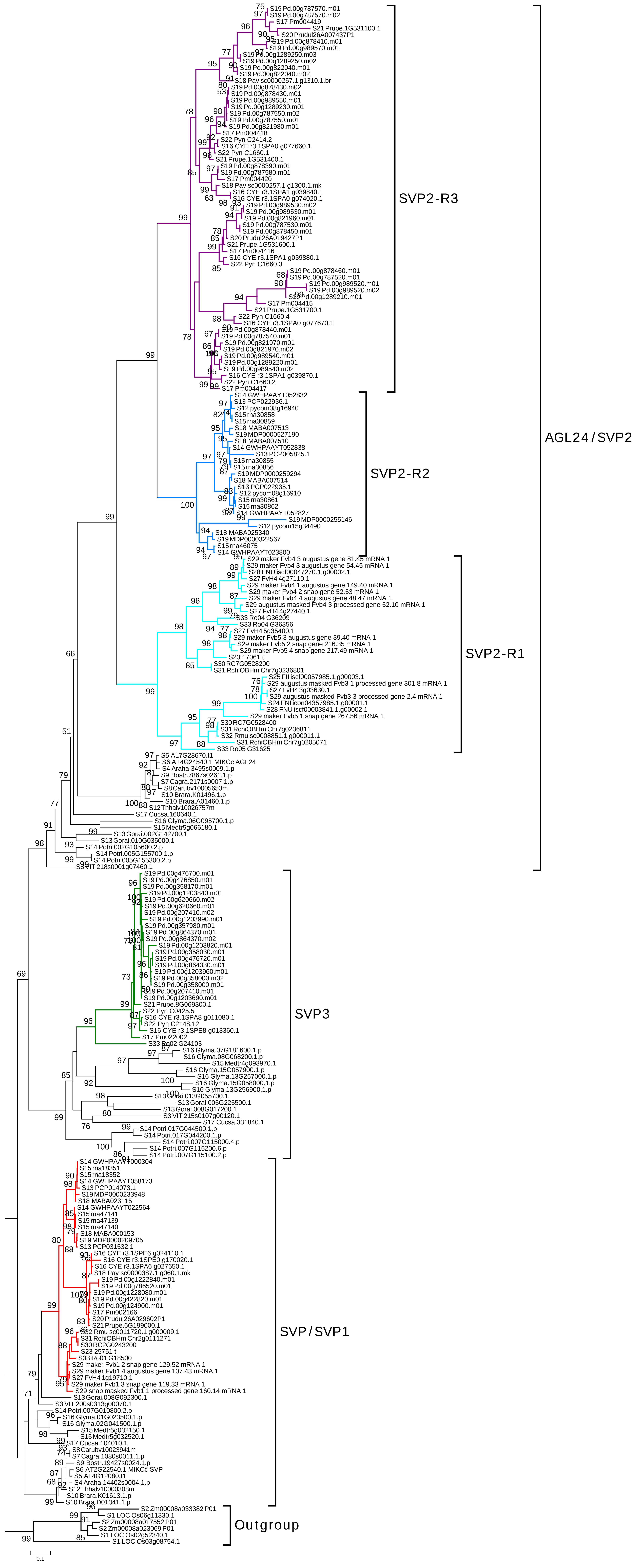

SVP2-R3
SVP2-R2
SVP2-R1
AGL24 / SVP2
SVP3
SVP / SVP1
Outgroup
0.1
